# Predictors of women’s utilization of primary health care for skilled pregnancy care in rural Nigeria

**DOI:** 10.1186/s12884-018-1730-4

**Published:** 2018-04-18

**Authors:** Friday Okonofua, Lorretta Ntoimo, Julius Ogungbangbe, Seun Anjorin, Wilson Imongan, Sanni Yaya

**Affiliations:** 1 Women’s Health and Action Research Centre, Km 11 Benin-Lagos Expressway, Benin City, Edo State Nigeria; 2 The University of Medical Sciences, Ondo City, Ondo State Nigeria; 3 The Federal University, Oye-Ekiti, Ekiti State Nigeria; 4 the Federal Bureau of Statistics, Abuja, Nigeria; 5 the Centre of Excellence in Reproductive Health Innovation (CERHI), University of Benin, Benin City, Nigeria; 6 the University of Ottawa, Ottawa, Canada

**Keywords:** Antenatal care, Delivery care, Primary health centres, Pregnant women, Edo State, Nigeria

## Abstract

**Background:**

Although Primary Health Care (PHC) was designed to provide universal access to skilled pregnancy care for the prevention of maternal deaths, very little is known of the factors that predict the use of PHC for skilled maternity care in rural parts of Nigeria - where its use is likely to have a greater positive impact on maternal health care. The objective of this study was to identify the factors that lead pregnant women to use or not use existing primary health care facilities for antenatal and delivery care.

**Methods:**

The study was a cross-sectional community-based study conducted in Esan South East and Etsako East LGAs of Edo State, Nigeria. A total of 1408 randomly selected women of reproductive age were interviewed in their households using a pre-tested structured questionnaire. The data were analyzed with descriptive and multivariate statistical methods.

**Results:**

The results showed antenatal care attendance rate by currently pregnant women of 62.1%, and a skilled delivery of 46.6% by recently delivered women at PHCs, while 25% of women delivered at home or with traditional birth attendants. Reasons for use and non-use of PHCs for antenatal and delivery care given by women were related to perceptions about long distances to PHCs, high costs of services and poor quality of PHC service delivery. Chi-square test of association revealed that level of education and marital status were significantly related to use of PHCs for antenatal care. The results of logistic regression for delivery care showed that women with primary (OR 3.10, CI 1.16–8.28) and secondary (OR 2.37, CI 1.19–4.71) levels education were more likely to receive delivery care in PHCs than the highly educated. Being a Muslim (OR 1.56, CI 1.00–2.42), having a partner who is employed in Estako East (OR 2.78, CI 1.04–7.44) and having more than five children in Esan South East (OR 2.00, CI 1.19–3.35) significantly increased the odds of delivery in PHCs. The likelihood of using a PHC facility was less for women who had more autonomy (OR 0.75, CI 0.57–0.99) as compared to women with higher autonomy.

**Conclusion:**

We conclude that efforts devoted to addressing the limiting factors (distance, costs and quality of care) using creative and innovative approaches will increase the utilization of skilled pregnancy care in PHCs and reduce maternal mortality in rural Nigeria.

## Background

The high rate of maternal mortality is a major public health concern in Nigeria. Several studies have shown that pregnant women in rural areas of the country are at greater risk of dying during pregnancy or childbirth as compared to those that live in urban areas [1, 2]. This is largely due to limited access to maternal health care by rural women as health facilities are often located far from where they live, with the result that they rely more on use of traditional sources of care or to no care at all [3, 4].

The Nigerian health care system is founded on a tripod of Primary Health Care, Secondary Health Care and Tertiary Health Care. In compliance with global recommendations for optimal maternal health care [5], Primary Health Care provides Basic Emergency Obstetrics Care (BEOC) comprising skilled delivery care, administration of antibiotics, manual removal of the placenta, removal of retained products of conception, assisted vaginal delivery possibly with a vacuum extractor, and basic neonatal care including neonatal resuscitation. By contrast, Secondary and Tertiary Health facilities (consisting of General and Teaching Hospitals) provide Comprehensive Emergency Obstetrics Care (CEOC) that consists of all BEOC services as well as caesarean section, safe blood transfusion services and the treatment of the sick baby. The Federal Ministry of Health of Nigeria specifically recommends Primary Health Care as the entry point to the health care system in order to generate universal health coverage for all citizens [6].

There are currently 33,000 Primary Health Centres (PHCs) located in 774 Local Government Areas (LGAs) with a minimum of ten wards per LGA in Nigeria. Each ward has a population of between 5000 to 10,000 persons, with each expected to have a PHC that provides immediate point of entry to the health care system for pregnant women seeking skilled pregnancy care. Thereafter, women with complications are referred for CEOC provided in Secondary and Tertiary care facilities [6, 7].

Despite this organized system, available evidence suggests considerable under-utilization of available PHC facilities for care by women seeking antenatal and intra-partum care in rural areas of the country [8–11]. Data from the Nigeria Demographic and Health Survey [12] indicates that in 2013 whereas 86.0% of urban women in the country received antenatal care from a skilled birth attendant (doctor or midwife), only 46.5% of rural women received such skilled antenatal service. During the same period, only 21.9% of rural women were delivered by a skilled birth attendant, compared to 61.7% of urban women. By contrast, a large proportion of rural women (77%) throughout the country delivered at home or in the homes of traditional birth attendants, compared to 37% of urban women.

Clearly, the tendency for rural women not to receive skilled pregnancy care has been recognized as one of the most important social determinants of the high rate of maternal mortality in Nigeria. Universal health coverage for maternal health is currently lacking in the country largely due to poor use of available and affordable services in PHCs [13]. Efforts to reverse this trend will greatly boost current efforts to improve maternal health, improve women’s access to skilled pregnancy care and prevent maternal deaths in the country.

Utilization of maternal healthcare services has been the subject of many descriptive and analytical studies in Nigeria [4, 14–19]. However, most studies did not disaggregate the analysis of the determinants by level of care (primary, secondary or tertiary) probably because many of the studies used secondary sources such as the National HIV/AIDS and Reproductive Health Surveys and Nigeria Demographic and Health surveys. Thus, little empirical evidence exists from primary sources on the reasons why women use or do not use primary healthcare facilities in Nigeria for maternal health care and the individual-level predictors of use or non-use. Analyses of utilization of primary health care facilities in Nigeria have been limited in their focus such as description of interventions [20, 21] quality of care and patients or community satisfaction, knowledge and use of partograph, adequacy of resources and providers, adequacy of antenatal care [9, 22–27]. Egbewale and Odu [8] described perceptions about PHC services and utilization highlighting reasons for use and non-use but this study was not specific on maternal healthcare. Ejembi et al. [11] presented utilization of PHC facilities for maternal care in two rural Hausa communities but it was a descriptive study limited to identifying the determinants.

The current analysis extends these past studies by engaging descriptive and analytical techniques to describe reasons for use and non-use of PHC facilities for maternal care and the individual-level predictors. The objective of this study was to identify the factors that lead pregnant women to use or not use existing primary health care facilities for antenatal and delivery care in two LGAs of Edo State. We believe the results would be useful for the design of interventions to improve women’s access to existing skilled pregnancy care at the primary health care facilities and reduce maternal mortality in the LGAs, with potential for scale up for greater impact throughout the country.

### Conceptual framework

This research is anchored on a behavioural model of health service use proposed by Andersen and Newman [28]. The framework was first developed in the 1960s and was designed to understand the conditions that predict utilization of health care services in the US. However, the framework has been adapted to research outside the US as described in a systematic review of studies using the model between 1998 and 2011 [29]. The model views utilization of health services as a form of individual behavior that is determined by individual characteristics of people which are influenced by societal and health systems determinants. The societal determinants (technology and societal norms such as health care financing) affect the individual determinants directly and through the health system determinants (resources - volume and distribution of labour and capital for health care, and organization - patient access to the medical care system, and structure- what happens after entry into the system). The individual characteristics that predict use of health services are classified into three: predisposition of an individual to use health services (predisposing factors), ability to secure services (enabling factors) and illness level (need factors). Predisposing factors include demographic characteristics such as age, sex, marital status, past illness, social structure (education, race, occupation, family size, ethnicity, religion, and residential mobility) and beliefs (values about health and illness, attitudes toward health services/providers, knowledge about disease). Previous research shows that the demographic characteristics of individuals predict their health behavior. For instance, being in a marital union is associated with better health and health-related behavior [30–32]. The past illness factor suggests that past experience of pregnancy and childbirth, parity, and experience of using a health care facility may affect the use of a primary health care facility for maternal care [28].

The enabling factors refer to the means available to individuals to achieve a need to use a health service. Enabling factors include family resources (income, level of health insurance coverage, or other source of third-party payment, type of regular source of care, the nature of that regular source of care, and accessibility of the source) and community characteristics (ratio of health personnel and facilities to population in a community, price of health services, region, urban-rural location). This implies that women’s ability to use maternal health facilities will depend on the availability of such facilities and their possession of the means to access the facilities.

The need factors include perceived illness or the probability of its occurrence by the individual or her family (disability, symptoms, diagnosis, general state –such as number of days during which the individual is unable to do her usual work such as house chores, care of children, experience of symptoms, self-report of general state of health), and evaluation of the condition (symptoms and diagnosis – attempts to get at the actual illness and a clinical assessment of the severity). According to Andersen and Newman [28], these factors represent the most immediate determinants of health service utilization. The need component suggests that the utilization of maternal health services can be influenced by a woman’s perception of the relative importance of modern health care services versus traditional methods of care. Added to this is a woman’s perception and understanding of pregnancy complications and her desire to deliver safely and attain a healthy newborn baby.

## Method

### Study communities

The study was a cross-sectional community-based study conducted in Esan South East and Etsako East LGAs in Edo State in southern Nigeria. Edo State is one of Nigeria’s 36 federating States located in the South-South geo-political zone of the country. Both LGAs are located in the rural and riverine areas of the state, adjacent to River Niger, with Estako East in the northern part of the Edo State part of the river, while Esan South East is in the southern part. Administratively, each LGA comprises of 10 wards, with several communities located in each ward. The two LGAs have a total population of 313,717 persons, with Esan South East accounting for 167,721 and Etsako East LGA accounting for 145,996. The principal sources of maternity care in the two LGAs are Primary Health Centres (PHCs). However, Esan South East LGA has one General Hospital in Ubiaja (headquarters of the LGA) while Etsako East has one General Hospital in Agenebode (the LGA administrative headquarters) and another in nearby Fugar City. Several private hospitals also exist in both LGAs that offer maternal and child health services of various degrees of quality. These public and private facilities are used as additional to the existing PHCs or for referral maternal health services.

### Sampling technique, data source and study population

The study was drawn from a survey conducted in July–August, 2017 as part of baseline data for the design of an on-going intervention research project to increase the access of rural women to skilled pregnancy care in 20 communities in Esan South East and Etsako East LGAs. A sample size of 1450 was derived for the project using the following formula:

$$ {\mathrm{n}}_1=\left\{\left[{\mathrm{p}}_1{\mathrm{q}}_1+{\mathrm{p}}_{\mathrm{o}}{\mathrm{q}}_{\mathrm{o}}\right]\right)\ {\left({\mathrm{Z}}_{\upalpha /2}+{\mathrm{Z}}_{\upbeta}\right)}^2\Big\}/{\left({\mathrm{p}}_1-\mathrm{po}\right)}^2 $$

p_0_ = utilization of PHC for maternal and perinatal in the control arm (assumed to be − 5 reduction in the prevalence in the experiment site).

p_1_ = utilization of PHC for maternal and perinatal care in the experimental arm.

*z*_*α*_ = Two-sided standard normal variate at 95% level of significance = 1.96.

*z*_*β*_ = Statistical power at 80% = 0.84;

$$ {\mathrm{n}}_1={\mathrm{n}}_2 $$

n_1_ = no of study participants in the experimental group.

n^2^ = no of study participants in the control group.

We assume 50% since there is no literature from the geographical location of the study which reported the prevalence of utilization of PHCs for maternal and perinatal health care. Thus:

$$ {\displaystyle \begin{array}{l}{\mathrm{n}}^1=\left(0.50x1-0.50+0.45x1-0.45\right)\ {\left(1.96/2+0.84\right)}^2/{\left(0.50-0.45\right)}^2\\ {}\left(0.25+0.2475\right)\ (3.3124)/0.0025\\ {}{\mathrm{n}}^1=659\\ {}{\mathrm{n}}^2=659\end{array}} $$

Total sample size = 1318.

10% adjustment for non-response = 132.

Total = 1450 (725 respondents in the experiment LGA and 725 in the control LGA).

A multi-stage sampling technique was used to select communities and the respondents in each LGA. Two rural LGAs (Esan South East and Etsako East) were purposively selected from the 18 LGAs in Edo state. Each LGA is divided into 10 health/administrative wards, with each ward made up of communities. Twenty (20) communities were selected purposively for the study – 10 from each LGA. Five communities per LGA were selected from where a PHC facility is located, while the other five communities were those in areas where there are no PHC facilities. Particular communities were selected systematically. In Esan South East, PHCs are located in 24 communities, while 75 communities have no PHCs. In contrast, in Etsako East, PHC facilities are located in 27 communities, while 15 communities had no PHC facilities. A sample interval was generated and 5 communities were selected systematically from a list of communities with PHCs and 5 from communities without a PHC in each LGA.

Within communities, households were listed and the number of women of reproductive age in each household was obtained. All eligible women in each household were interviewed. The eligibility criteria were age 15–45 years, ever married, currently pregnant or have had a birth in the 5 years preceding the survey. In Etsako, 1487 households were listed and there were 1051 women of reproductive age in the households. Out of the 1051, 707 eligible women were interviewed with a non-response rate of 2.5%. In Esan South East, 1975 households were listed with 1084 women of reproductive age, 701 eligible women were interviewed with 3.3% non-response. A total of 1408 eligible women were interviewed in the two LGAs, with 2.9% overall non-response rate.

A questionnaire prepared by the investigators was used for data collection. The instrument (questionnaire) was pretested in a rural community with similar characteristics with the study locations. The questionnaire consisted of five sections. Section one contained the respondents’ socio-demographic characteristics; section 2 was related to partners’ and other family characteristics; section 3 contained questions on the respondents’ reproductive history; section 4 was on antenatal, intrapartum and postnatal care experience for current pregnancy and births in the preceding 5 years; while section 5 contained questions on reasons for use and non-use of PHCs for maternal and child care.

Drawing from the literature, some reasons for use and non-use of a PHC facility for maternal health care were provided as multiple response options. The respondents selected as many options as are applicable to them. The following options of reasons for use were provided: cost not too much, no charges, facility is always open, provider is available, facility not far from my house, good quality service (subjective opinion of the respondent on the care provided in PHC facilities), husband wanted it, family wanted it, adequate security, other (specify). The reasons for non-use provided in the questionnaire were: cost too much, facilities not open, no provider in the facility, facility too far, no transport to facility, poor quality service (subjective view of the respondent on the care received), husband did not allow, family did not allow, no time because baby came suddenly, my culture forbids, no security, and other (specify). Additional reasons for use and non-use shown in the result tables were drawn from the other (specify) category.

The questionnaire was entered into Computer-Assisted Personal Interviewing (CAPI) using a Census and Survey Processing System (CSPro) software (CSentry). CSPro is a public domain software package developed by the US Census Bureau and ICF International. It is widely used for entering, editing, tabulating and disseminating census and survey data. The software runs on the Microsoft windows and Android families of operating systems [33]. Thus, instead of the paper and pen interviewing, the CAPI facilitated accuracy and speed in the data collection process. The questionnaire was administered through face-to-face interviewing by trained field assistants. The questions were fielded in English or in Pidgin English as appropriate, since all women in both communities either understand English or Pidgin English.

### Variables and measures

The dependent variable for antenatal care was place of antenatal care: PHC facility coded 1 and other facilities/home coded 0. The dependent variable for delivery care was place of delivery; use of a PHC facility was coded 1 while other facilities and home was coded 0. Drawing on the model of health services utilization and past studies on utilization of facilities for maternal care in Nigeria, the following independent variables were included in the analyses: age, highest level of education, exposure to media, religion, employment (working and not working), marital status (married, living together, widowed, divorced, and separated), age at marriage, partner’s age, partner’s highest education, partner’s employment, who pays for respondent’s health care (respondent alone, husband alone or others, and respondent with husband), level of autonomy (less, more and much), number of living children, education difference between respondent and partner (both have no education, husband more educated than wife, wife more educated than partner, and same level of education but not none), and LGA.

Exposure to media was generated with frequency of listening to radio and watching television. The response options were everyday, at least once a week, less than once a week, and not at all. The responses were aggregated to generate a three-category measure of exposure to the media: high, moderate and no exposure. No exposure refers to neither listens to radio nor to television at all. An index for autonomy was generated with responses to 6 questions on ownership of land/house, participation in household decisions on respondent’s health, major purchases, daily purchases, visits to family and friends, food to be cooked. Ownership of land or house was included because women in the study location are allowed to own land or a house if they wish. The response options were respondent alone, husband alone, respondent and husband, and others. These responses were further collapsed into two categories labeled respondent alone or respondent with husband indicating autonomy (coded 1) husband alone & others indicating no autonomy (coded 0) for participation in decisions. Ownership of land/house was categorized into respondent alone or with husband indicating autonomy (coded 1), owns no land/house and husband alone owns as no autonomy (coded 0). Using principal component analysis, a three-category index of autonomy was generated (less, more and much); scale reliability coefficient was 0.70. A response of 0 in all 6 questions and 1 in 1–2 questions was less autonomy; response of 1 in 3–4 questions was more autonomy, and a response of 1 in 5–6 questions was much autonomy. Women’s participation in household decision-making, her health care, mobility, and ownership of land among others have been used in many previous studies as measures of women’s autonomy [34–36].

### Data analysis

The current analysis is based on antenatal care for currently pregnant respondents, and delivery or intrapartum care for the most recent births by the respondents. The data were extracted from the CAPI devise, cleaned and analyzed with STATA 12 for windows. To describe the characteristics of the respondents, univariate analysis using percentages and summary statistics was conducted. Reasons for use and non-use of a PHC facility for maternal care (antenatal care for current pregnancy and delivery care for the most recent births) were elicited from multiple response options. The results are presented as number of responses and percentages for each of the specified options and a few additional categories drawn from the other (specify) option. To compare proportions for each reason for use and non-use of a PHC for antenatal and delivery care between the two LGAs, a two-sample test of proportions was conducted.

Due to the small sample size for currently pregnant respondents who are receiving antenatal care (*n* = 175), a bivariate analysis using chi-squared test was conducted to test the relationship between the use of a PHC facility for antenatal care for currently pregnant respondents and selected characteristics of the respondents. Selection of the characteristics was based on their potential theoretical and practical influence on the use of a PHC facility for antenatal care. Binary logistic regression was conducted to determine the predictors of PHC facility use for delivery care during the most recent births by the respondents.

Some of the independent variables were re-coded for the chi-squared test and the multivariate analysis because of zero or few cases in some categories. The variables that were re-coded for the chi-squared test were age, level of education, religion (traditionalist and others were dropped because they had few cases and cannot be merged with any other category), marital status, (widowed, divorced, and separated were dropped because of few cases even after collapsing the three as formerly married). Others were partner’s age, partner’s level of education, payment for respondent’s healthcare. In the multivariate analysis for delivery care, traditional and other religions were dropped due to few cases; age and partner’s age was entered as a continuous variables. The variables included in the logistic regression model were either significant in a bivariate logistic regression model at 0.05 or 0.10 level of significance or conceptually important drawing from the behavioral model of health services utilization, past studies and the authors’ knowledge of the study population. A Wald test was also conducted to test whether the explanatory variables in the logit model are simultaneously equal to zero. The test result was significant indicating that including these variables creates a statistically significant improvement in the fit of the model. The results of the logistic regression are presented as odds ratio (OR) with 95% confidence interval for the entire study population and for each LGA. Statistical significance for all the statistical analysis was set at 0.05.

## Results

### Characteristics of the study population

The study population is described in Table 1. The mean age of the respondents was 30 years with a standard deviation (SD) of 6.9 years. The mean age in Esan South East (31 years) was slightly higher than the overall average whereas in Etsako East it was below the average (29 years). Most respondents had attained primary and secondary education, but in Etsako East, slightly more than half of the respondents had primary education unlike in Esan South East where the majority (45.1%) had secondary education. Exposure to media (listening to the radio and watching television) was moderate in both LGAs. Most respondents were Christians of non-Catholic affiliation. In both LGAs, the majority of the respondents were employed. An examination of the details of occupation showed that most were self-employed in the low-wage informal sector as traders, farmers, tailors, and hair stylists (not shown in the table).

**Table 1:** Percent distribution of the respondents by personal, family and reproductive characteristics by LGA

Characteristic	All	Esan SE	Etsako East
Number of respondents	1408	701(49.8)	707(50.2)
Personal Characteristics			
Age			
Mean	30(SD 6.9)	31.5(SD 6.9)	28.6(SD 6.7)
16–19	64(4.5)	21(3.0)	43(6.1)
20–24	260(18.4)	87(12.4)	173(24.5)
25–29	354(25.1)	167(23.8)	187(26.4)
30–34	303(21.5)	175(24.9)	128(18.1)
25–39	249(17.7)	131(18.7)	118(16.7)
40–47	178(12.6)	120(17.1)	58(8.2)
Education			
No Education	206(14.6)	76(10.8)	130(18.4)
Primary	617(43.8)	253(36.1)	364(51.5)
Secondary	503(35.6)	316(45.1)	186(26.3)
Higher	83(5.9)	56(8.0)	27(3.8)
Exposure to media			
High exposure	420(29.8)	216(30.8)	204(28.8)
Moderate exposure	666(47.3)	392(55.9)	274(38.8)
No exposure	322(22.9)	93(13.3)	229(32.4)
Religion			
Catholic	369(26.2)	123(17.5)	246(34.8)
Other Christian	884(62.8)	551(78.6)	333(47.2)
Islam	145(10.3)	21(3.0)	124(17.6)
Traditionalist	8(0.6)	5(0.7)	3(0.4)
Other	1(0.1)	1(0.1)	0(0.0)
Employment			
Not working	287(20.4)	127(18.1)	160(22.6)
Working	1121(78.6)	574(81.9)	547(77.4)
Marital Status			
Married	926(65.8)	432(61.6)	494(69.9)
Living together	447(31.7)	248(35.4)	199(28.2)
Widowed	15(1.1)	11(1.6)	4(0.6)
Divorced	1(0.1)	1(0.1)	0(0.0)
Separated	19(1.3)	9(1.3)	10(1.4)
Age at marriage			
Mean (SD)	21.0(3.9)	21.8(4.2)	20.2(3.5)
14–17	235(16.7)	90(12.8)	145(20.5)
18–24	875(62.1)	415(59.2)	460(65.1)
25–29	249(17.7)	159(22.7)	90(12.7)
30–39	49(3.5)	37(5.3)	12(1.7)
Partner/Family Characteristics			
Partner’s age			
Mean(SD)	39.4(9.2)	40.2(8.6)	38.5(9.6)
18–24	36(2.6)	13(1.9)	23(3.3)
25–29	142(10.2)	46(6.7)	96(13.7)
30–34	235(16.9)	104(15.1)	131(18.6)
35–39	255(18.3)	133(19.3)	122(17.3)
40–44	283(20.3)	160(23.2)	123(17.5)
45–49	239(17.2)	126(18.3)	113(16.1)
50–54	125(9.0)	73(10.6)	52(7.4)
55–82	78(5.6)	35(5.1)	43(6.1)
Partner’s Education			
No Education	104(7.4)	62(8.8)	42(5.9)
Primary	348(24.7)	155(22.1)	193(27.3)
Secondary	737(52.3)	372(53.1)	365(51.6)
Higher	219(15.6)	112(16.0)	107(15.1)
Spousal education Difference			
Both have none	55(3.9)	24(3.4)	31(4.4)
Husband more	622(44.2)	239(34.1)	383(54.2)
Wife more	127(9.0)	91(13.0)	36(5.1)
Same but not none	604(42.9)	347(49.5)	257(36.3)
Partner’s Employment			
Not working	82(5.8)	49(7.0)	33(4.7)
Working	1326(94.2)	652(93.0)	674(95.3)
Autonomy			
Less	507(36.0)	243(34.7)	264(37.3)
More	465(33.0)	220(31.4)	245(34.7)
Much	436(31.0)	238(33.9)	198(28.0)
Payment for respondent’s health care			
Respondent alone	107 (7.6)	68(9.7)	39(5.5)
Partner alone	1140(81.0)	533(76.0)	607(85.9)
Respondent & partner	144(10.2)	92(13.1)	52(7.4)
Other	17(1.2)	8(1.1)	9(1.3)
Reproductive Characteristics			
Number of living children			
0–2	456(34.1)	230(33.2)	226(35.0)
3–4	415(31.0)	218(31.5)	197(30.5)
5+	466(34.9)	244(35.3)	222(34.4)
Currently pregnant			
Yes	277(19.7)	81(11.6)	196(27.7)
No	1120(79.5)	612(87.3)	508(71.9)
Unsure	11(0.8)	8(1.10	3(0.4)
Current Pregnancy (*n* = 277)			
Receiving antenatal care (ANC)			
Yes	172(62.1)	51(63.0)	121(61.7)
No	105(37.9)	30(37.0)	75(38.3)
Place of ANC			
Other govt. facility	11(6.3)	7(13.7)	4(3.2)
PHC	145(82.9)	39(76.5)	106(85.5)
Private Hospital	16(9.1)	4(7.8)	12(9.7)
Other	3(1.7)	1(2.0)	2(1.6)
Most recent birth (*n* = 1314)			
Antenatal care^a^			
Yes	168(91.8)	67(90.5)	101(92.7)
No	15(8.2)	7(9.5)	8(7.3)
Place of Antenatal care			
PHC	146 (84.9)	56 (84.9)	90 (84.9)
Other govt. hospital	14 (8.1)	5 (7.6)	9 (8.5)
Private hospital	10 (5.8)	5 (7.6)	5 (4.7)
Home	2 (1.2)	0 (0.0)	2 (1.9)
Place of delivery			
Other govt. facility	158(12.0)	96(13.9)	62(10.0)
PHC	612(46.6)	365(52.7)	247(39.8)
Private Hospital	218(16.6)	136(19.6)	82(13.2)
At home/other	326(24.8)	96(13.8)	230(37.0)

Note: ^a^most of the respondents did not respond to the question on antenatal care for their most recent birth. The reported percentage of no antenatal care should be interpreted with caution

Most respondents were in marital union. Slightly above 30% of those in union were living together with a partner (informal union). The proportion living together with a partner was higher in Esan South East (35.4%) than in Etsako East (28.2%). Mean age at first marriage was 21 years. The mean age of their partners was 39.4 years, slightly above 50% of the partners attained secondary education and most of them worked in formal and informal employments. With regard to gap in the level of education between the respondents and their partners, 44.2% had partners who were more educated, while 42.9% had the same level of education with their partners. In Esan South East, most of the respondents had same level of education with their partners (49.5%) whereas the majority in Etsako East had partners who were more educated (54.2%). Respondents with less autonomy were in higher proportion in both LGAs; and for the majority of the respondents, partner alone pay for their health care.

Close to 20% (277) of the respondents were currently pregnant with a higher proportion in Etsako East. Among those who were currently pregnant, 62.1% were receiving antenatal care, and out of those who were receiving care, most of them were using a PHC facility. With regard to delivery care for the most recent births, 1314 respondents had a recent birth and only few reported receiving any antenatal care. Among the few, most (84.9%) received antenatal care in a PHC facility. Response to the question on place of delivery showed that 46.6% delivered in a PHC facility, while close to 25% delivered at home and with a Traditional Birth Attendant (TBA). In Esan South East, many respondents delivered in a PHC facility (52.7%) and in Etsako East, close to 40% delivered in a PHC. Unlike in Esan South East where 13.8% delivered at home and with a TBA, 37% of the respondents in Etsako East had their most recent births at home and with a TBA.

### Reasons for use and non-use of a PHC facility

The respondents were asked to provide reasons why they used or did not use a PHC facility for antenatal care (current pregnancy) and delivery care for their most recent births.

### Reasons for use

Reasons for use of PHC for antenatal and delivery care are presented in Table 2. The most frequently mentioned reasons for using a PHC facility for antenatal care by currently pregnant respondents were that the facility is close to their residence (27.2%), good quality service (19.9%), and provider is available (14.1%). The least mentioned reasons were no charges (0.6%), and adequate security (0.9%). This pattern of response for antenatal care was similar across the two LGAs. The two-sample test of proportions for reasons for using a PHC for antenatal care indicates that the proportions differed significantly between the two LGAs. Particular reasons that were significantly different were cost too much, providers not available, facility not far from respondent’s residence, good quality service, husband wanted it, family wanted it, adequate security, and other reason.

**Table 2:** Percent distribution of reasons for using a PHC facility for antenatal and delivery - Number of responses (%)

	Antenatal care			Delivery care		
Reason	All	Esan South East LGA	Etsako East LGA	All	Esan South East LGA	Etsako East LGA
	(*n* = 533)	(*n* = 157)	(*n* = 376)	(*n* = 2294)	(*n* = 1303)	(*n* = 991)
Cost not too much	79(14.8)	24(15.3)	55(14.6)	386(16.8)	228(17.5)	158(15.9)
No charges	3(0.6)	2(1.3)	1(0.3)	20(0.9)	16(1.2)	4(0.4)
Facility is always open	43(8.1)	17(10.8)	26(6.9)	236(10.3)	161(12.4)	75(7.6)
Provider are available	75(14.1)	24(15.3)	51(13.6)	375(16.3)	220(16.9)	155(15.6)
Facility Not far from my house	145(27.2)	31(19.7)	114(30.3)	465(20.3)	208(16.0)	257(25.9)
Good quality service	106(19.9)	37(23.6)	69(18.4)	451(19.7)	259(19.9)	192(19.4)
Husband wanted it	54(10.1)	11(7.0)	43(11.4)	193(8.4)	96(7.4)	97(9.8)
Family wanted it	7(1.3)	5(3.2)	2(0.5)	63(2.7)	51(3.9)	12(1.2)
Adequate security	5(0.9)	5(3.2)	0(0.0)	43(1.9)	37(2.8)	6(0.6)
Baby’s health/safety	7(1.3)	0 (0.0)	7(1.9)	14(0.6)	0(0.0)	14(1.4)
No other facility	–	–	–	10(0.4)	9(0.7)	1(0.1)
^a^ Other	9(1.7)	1(0.6)	8(2.1)	38(1.7)	18(1.4)	20(2.0)
Test of proportions	*p* = 0.0000			*p* = 0.0070		

^a^Other includes reasons such as nothing, nice matron, works in a PHC, relative works there, to get birth certificate, and it is not necessary among others

Reasons for using a PHC facility for delivery care were similar to the reasons for using a PHC facility for antenatal care: facility close to residence, good quality care and provider is available were the most frequently mentioned reasons. The least reasons were no charges, baby’s safety/health and no other facility. The only variation for the LGAs was in Esan South East where good quality service was the most frequently mentioned reason, but Etsako East followed the general pattern of facility near residence, good quality care and provider available. Reasons for using a PHC for delivery care varied significantly between the two LGAs. Specific reasons that differed significantly were cost not too much, no charges, facility is always open, providers are available, facility not far from respondent’s residence, good quality service, family wanted it and adequate security.

### Reasons for non-use

The distribution of reasons for non-use of a PHC facility is shown in Table 3. Of the 97 responses elicited from currently pregnant respondents, no provider in the facility (17.5%), poor quality service (17.5%), and facility not open (12.3%) were the most commonly mentioned reasons. The least mentioned reasons were preference for home delivery/TBA, family did not allow and no PHC facility. Reasons such as my culture forbids and no security were not mentioned at all. In Esan South East, the most frequently mentioned reasons were poor quality service (47.6%), no provider in the facility (14.3%), and husband did not allow (14.3%), whereas in Etsako no provider in the facility featured prominently, followed by poor quality service and husband did not allow. There was a statistically significant difference between LGAs in all the reasons for non-use of a PHC for antenatal care except for husband and family wanted it.

**Table 3:** Percent distribution of reasons for non-use of a PHC facility for antenatal and delivery care - Number of responses (%)

	Antenatal care			Delivery care		
Reason	All	Esan South East LGA	Etsako East LGA	All	Esan South East LGA	Etsako East LGA
	(*n* = 97)	(*n* = 21)	(*n* = 76)	(*n* = 532)	(*n* = 243)	(*n* = 289)
Cost too much	7(7.2)	0(0.0)	7(9.2)	48(9.0)	14(5.8)	34(11.8)
Facility not open	12(12.3)	0(0.0)	11(14.5)	46(8.6)	4(1.6)	42(14.5)
No provider in the Facility	17(17.5)	3(14.3)	14(18.4)	64(12.0)	26(10.7)	38(13.1)
Facility too far	8(8.2)	2(9.5)	6(7.9)	62(11.7)	33(13.6)	29(10.0)
No transport to Facility	5(5.2)	0(0.0)	5(6.6)	21(3.9)	8(3.3)	13(4.5)
Poor quality service	17(17.5)	10(47.6)	7(9.2)	104(19.5)	67(27.6)	37(12.8)
Husband did not allow	10(10.3)	3(14.3)	7(9.2)	27(5.1)	10(4.1)	17(5.9)
Family did not allow	4(4.1)	2(9.5)	2(2.6)	9(1.7)	5(2.1)	4(1.4)
No time because baby came suddenly	–	–	–	33(6.3)	13(5.3)	20(6.9)
My culture forbids	0(0.0)	0(0.0)	0(0.0)	5(0.9)	0(0.0)	5(1.7)
No Security	0(0.0)	0(0.0)	0(0.0)	2(0.4)	1(0.4)	1(0.3)
No PHC facility	4(4.1)	0(0.0)	4(5.3)	20(3.8)	1(0.4)	19(6.6)
Prefer home delivery/TBA	2(2.1)	0(0.0)	2(2.6)	–	–	–
Choice	–	–	–	5(0.9)	5(2.1)	0(0.0)
Had complications	–	–	–	2(0.4)	2(0.8)	0(0.0)
Dislike PHC	–	–	–	8(1.5)	4(1.6)	4(1.4)
Referred	–	–	–	5(0.9)	5(2.1)	0(0.0)
^a^Other	12(12.4)	1(4.8)	11(14.5)	71(13.3)	45(18.5)	26(9.0)
Test of proportions	*p* = 0.0001			*p* = 0.3889		

^a^Other includes reasons such as dislike for injection/hospital, no money, nothing, and fear among others

The dominant reasons for not using a PHC for delivery care in the most recent births of the respondents were poor quality service (19.5%), no provider in the facility (12.0%), and facility is too far (11.7%). The least reasons were my culture forbids and no security. Distribution of responses in Esan South East showed poor quality service, facility too far and no provider as the common reasons whereas facility not open, no provider and poor quality service were the most frequently mentioned reasons in Etsako East. The reasons for non-use of a PHC for delivery care were not found to be statistically different between the LGAs. However, specific reasons such as cost too much, facility not open, poor quality service, and culture forbids were significantly different.

### Factors related to use of a PHC for antenatal care

The result of chi-squared test to examine the relationship between use of a PHC for antenatal care and selected characteristics of the respondents who were pregnant during the survey is presented in Table 4. The relationship between place of antenatal care and respondent’s level of education was statistically significant. About 61% of those who had no education or primary education used a PHC compared to 38.6% of those who attained secondary and higher levels of education. This corresponds to a difference of 22.8%. Marital status was significantly associated with the use of a PHC facility for antenatal care. Close to 60% of the respondents who were married compared to 40.3% of those living together with a partner were using a PHC facility for antenatal care for their current pregnancy.

**Table 4:** Association between Place of Antenatal Care and Selected Respondents’ Characteristics

Characteristic	PHC facility	Other	Pearson Chi2/*p*-value
LGA			
Esan South East	39(26.9)	12(40.0)	(1) = 2.0668
Etsako East	106(73.1)	18(60.0)	*p* = 0.151
Age			
16–30	108(74.5)	24(80.0)	(1) = 0.4083
31–47	37(25.5)	6(20.0)	*p* = 0.523
Level of education			
No education/primary	89(61.4)	11(36.7)	(1) = 6.1988
Secondary/higher	56(38.6)	19(63.3)	*p* = 0.013
Exposure to media			
High exposure	37(25.5)	10(33.3)	
Moderate exposure	65(44.8)	15(50.0)	(2) = 2.2397
No exposure	43(29.7)	5(16.7)	*p* = 0.326
Religion			
Catholic	32(22.2)	12(40.0)	
Other Christian	74(51.4)	13(43.3)	(2) = 4.3749
Islam	38(26.4)	5(16.7)	*p* = 0.112
Employment			
Not working	55(37.9)	7(23.3)	(1) = 2.3154
Working	90(62.1)	23(76.7)	*p* = 0.128
Marital status			
Married	86(59.7)	26(86.7)	(1) = 7.8589
Living together	58(40.3)	4(13.3)	*p* = 0.005
Age at marriage			
14–17	24(16.5)	2(6.7)	
18–24	100(69.0)	23(76.7)	
25–29	17(11.7)	4(13.3)	(3) = 1.9270
30–39	4(2.8)	1(3.3)	*p* = 0.588
Partner’s age			
18–29	41(28.3)	6(20.0)	
30–34	29(20.0)	6(20.0)	
35–39	28(19.3)	7(23.3)	
40–44	25(17.2)	8(26.7)	(4) = 2.4716
45–82	22(15.2)	3(10.0)	*p* = 0.650
Partner’s level of education			
None/primary	36(24.8)	5(16.7)	(1) = 0.9228
Secondary/higher	109(75.2)	25(83.3)	*p* = 0.337
Payment for respondents health care			
Husband alone/others	120(82.8)	29(96.7)	(1) = 3.8010
Respondent alone/with husband	25(17.2)	1(3.3)	*p* = 0.051
Autonomy			
Less	72(49.7)	12(40.0)	
More	34(23.4)	10(33.3)	(2) = 1.4493
Much	39(26.9)	8(26.7)	*p* = 0.484
Number of living children			
0–2	56(53.9)		
3–4	28(26.9)	15(65.2)	
5+	20(19.2)	4(17.4)	(2) = 1.1485
		4(17.4)	*p* = 0.563
Spousal education difference			
Same but not none	60(41.4)	15(50.0)	
Wife more	6(4.1)	2(6.7)	
Husband more	61(42.1)	11(36.6)	(3) = 1.6735
Both none/either none	18(12.4)	2(6.7)	*p* = 0.643

### Predictors of use of PHC for delivery care

Results of the logistic regression model predicting the factors associated with utilization of a PHC for delivery care are presented in Table 5. Some of the predictors were significantly associated with the use of a PHC facility for delivery care. Compared to the respondents who attained higher education (post-secondary), those who attained secondary (OR 2.37, CI 1.19–4.71), and primary education (OR 3.10, CI 1.16–8.28) were significantly more likely to use a PHC facility for delivery care. The odds of using a PHC for delivery care were significantly higher among Muslim respondents than Catholics (OR 1.56, CI 1.00–2.42). Respondents who had more autonomy were significantly less likely than those who had less autonomy to use a PHC facility for delivery care (OR 0.75, CI 0.57–0.99). The use of a PHC facility for delivery care in a respondent’s most recent birth was 61% more likely for respondents who have 5 or more living children relative to those who had 0–2 children.

**Table 5:** Logistic Regression model predicting the likelihood of using a PHC facility for delivery care

Variable	Odds Ratio (95% Confidence Interval)		
	All respondents	Esan South East	Etsako East
Age	0.96(0.94–0.99)*	0.97(0.93–1.01)	0.97(0.93–1.01)
Education			
Higher (Ref)	1.00	1.00	1.00
Secondary	2.37(1.19–4.71)*	1.69(0.67–4.26)	2.74(0.90–8.32)
Primary	3.10(1.16–8.28)*	2.17(0.53–8.88)	3.32(0.75–14.6)
No Education	2.36(0.70–7.93)	1.69(0.30–9.54)	2.35(0.38–14.2)
Exposure to media			
High (Ref)	1.00	1.00	1.00
Moderate	1.14(0.87–1.50)	1.14(0.79–1.65)	1.11(0.73–1.71)
None	1.35(0.96–1.91)	1.43(0.82–2.50)	1.30(0.82–2.08)
Religion			
Catholic (Ref)	1.00	1.00	1.00
Other Christian	1.20(0.91–1.58)	1.37(0.90–2.08)	1.02(0.70–1.48)
Islam	1.56(1.00–2.42)*	0.56(0.20–1.55)	1.87(1.13–3.10)*
Partner’s age	0.99(0.97–1.01)	0.97(0.94–1.00)	1.00(0.97–1.02)
Partner’s Education			
No Education (Ref)	1.00	1.00	1.00
Primary	0.76(0.33–1.76)	0.70(−.26–1.88)	1.07(0.20–5.80)
Secondary	0.99(0.34–2.83)	0.71(0.18–2.73)	1.45(0.20–10.5)
Higher	1.05(0.28–3.95)	0.49(0.08–3.04)	2.11(0.21–20.8)
Partner’s Employment			
Not working (Ref)	1.00	1.00	1.00
Working	1.62(0.95–2.77)	1.25(0.63–2.45)	2.78(1.04–7.44)*
Autonomy			
Less (Ref)	1.00	1.00	1.00
More	0.75(0.57–0.99)*	0.78(0.52–1.15)	0.76(0.51–1.15)
Much	1.11(0.83–1.47)	0.83(0.56–1.23)	1.51(0.98–2.33)
Number of children			
0–2 (Ref)	1.00	1.00	1.00
3–4	1.23(0.90–1.67)	1.18(0.76–1.83)	1.20(0.76–1.89)
5+	1.61(1.11–2.33)*	2.00(1.19–3.35)**	1.19(0.68–2.07)
Spousal Education Difference			
Both have none(Ref)	1.00	1.00	1.00
Husband more	1.12(0.35–3.59)	0.96(0.19–4.75)	1.08(0.13–8.65)
Wife more	1.84(0.68–5.00)	0.94(0.23–3.84)	2.23(0.41–11.9)
Same but not none	1.59(0.55–4.62)	1.06(0.25–4.51)	1.75(0.25–11.8)
LGA			
Esan South East (Ref)			
Etsako East	0.55(0.42–0.71)***		

*p < 0.05 **p < 0.01 ***p < 0.001

There was a statistically significant difference between the two LGAs in the use of a PHC facility. Utilising a PHC for delivery care was less likely in Etsako East than in Esan South East (OR 0.55, CI 0.42–0.71). Thus, a separate analysis was conducted for each LGA to determine what differences there might be between the LGAs in the predictors of PHC facility use for delivery care. In Esan South East, only number of living children predicted use of a PHC facility for delivery care. Respondents who have 5 or more living children were more likely than those who had 0–2 children to use a PHC for delivery care in their most recent birth (OR 2.00, CI 1.19–3.35). In Etsako East, use of a PHC facility for delivery care was positively associated with Islamic religious affiliation compared to Catholics (OR 1.87, CI 1.13–3.10), and respondents whose partners worked were more likely to use a PHC facility than those whose partners did not work (OR 2.78, CI 1.04–7.44).

## Discussion

The study was designed to investigate why women use or do not use PHCs for antenatal and delivery care in rural parts of Edo State in Southern Nigeria. We based the study on the premise that PHCs offer the best opportunity for rural women in Nigeria to enter the health care system to receive the most optimal evidence-based and cost-effective access to skilled delivery care [37, 38]. PHCs are not only located closest to rural women within the current health care system in the country; they also provide opportunity for health workers to offer personalized care that address the cultural and social realities of rural women.

We used different approaches to determine why women use or do not use PHCs for antenatal and delivery care. First, we asked pregnant women and recently delivered women where they are receiving or previously received antenatal care in their previous pregnancies. The results were more accurate for currently pregnant women, while we believe that the difficulty with recall of antenatal events hindered the ability of recently delivered women to provide reliable answers to the question. With currently pregnant women, we found that a large proportion in both LGAs (> 60%) were receiving antenatal care in PHCs. This was not unexpected since the women were drawn from communities that have PHCs as their primary sources of health care.

We then asked women who received antenatal care from PHCs the reasons they choose the facilities. The most commonly proffered reasons (in order of frequency) were: facility near to place of residence, good quality service, cost not too much, and husband wanted it. On the other hand, when the 40% of women in both LGAs who had not received antenatal care in PHCs were asked the reasons why they did not, the most common reasons (in order of frequency) were: poor quality service, no provider in the facility, facility not open, facility too far, and costs too much.

Thus, it was evident that reasons for use and non-use of PHCs for antenatal were related to perceptions about distance of PHCs, quality of PHC service delivery (costs, availability of health personnel, etc.), and partners consent to use of the facilities.

As for skilled delivery care in PHCs, evidently only women who recently delivered could be relied on to provide information on this question. The result showed that more than 45.0% of women delivered in PHCs while up to 25% delivered at home or in the homes of traditional birth attendants. Reasons for use of PHCs for delivery (in order of frequency) included: nearness of PHC to place of residence, good quality service, costs not too much, provider available, and husband’s desire. By contrast, the reasons given by women for not using PHCs for delivery were: poor quality service, provider not in facility, facility too far, costs too much, and facilities not open. Some of these reasons for non-use of a PHC facility for antenatal and delivery care have been identified in previous studies in Nigeria and other countries [8, 11, 39, 40]. Sustainable interventions that will specifically address these recurrent reasons, particularly among rural populations are imperative if Nigeria would achieve her developmental targets on maternal health.

Our bivariate analysis to determine how the use of PHCs is related to various socio-demographic variables showed that only women’s education and marital status were associated with use of PHCs for antenatal care. Married women as well as women with no education and those with primary level education were more likely to use PHCs for antenatal care as compared to women living together with a partner and women with secondary and higher level education. Previous studies show that formal marital union is associated with better health seeking behavior than consensual union or cohabitation [30–32].

The results of binary logistic regression to determine the independent effects of socio-economic pre-disposing variables in predicting the likelihood of use of PHCs for delivery care showed that higher education reduced the odds of delivery in PHCs in both LGAs. By contrast, the odd of delivery in PHC was higher in Esan South East as compared to Etsako East. Also, being a Muslim and having a partner who was employed increased the odds of delivery in Estako East, but not in Esan South East, while having more than five existing children significantly predicted the likelihood of delivery in a PHC in Esan South East but not in Etsako East.

It was of interest that higher level education of women in this study was shown to reduce the likelihood of women utilizing PHCs for antenatal and delivery care. This is contrary to the results of several studies on maternal health services utilization in Nigeria and other parts of Africa [41–44] which suggest that women with higher level education are more likely to utilize available health facilities. While such studies have addressed maternal health service utilization overall, very few have addressed service utilization at PHCs in rural communities. We believe that our result on education is attributable to the fact that women commonly identified poor quality care as the reasons for non-use of services in PHCs. Our sub-analysis showed that women with higher level education (secondary and tertiary) were more likely to use other facilities and to report poor quality care as reasons for non-use of PHCs. This is further supported by data showing that women with higher autonomy (but not those with better educated partners) were more likely not to use PHCs for antenatal and delivery care. Better educated women and women with higher levels of education are more likely to ignore the services in PHCs on account of perceptions about quality and presumably opt for services in private clinics or in secondary/tertiary care facilities [39].

We therefore recommend that improved quality service delivery would be an important and critical intervention to increase the access of rural women to antenatal and delivery care in PHCs. In this regard, attention needs to be paid by policymakers and health providers to addressing physical distance of PHCs, improving PHC infrastructure, availability of health personnel, reliability of drugs and equipment supplies, constancy of opening times and reduction in costs of services. Innovations and creativity around transportation of women to PHCs when in labor, community support for costs alleviation such as health insurance, community health education, and linkages to higher level care through the development of an effectiveness referral system would build confidence in the use of PHCs for antenatal and delivery care among rural women.

Contrary to our expectation, cultural preference for home births did not significantly feature as a reason for non-use of PHCs for antenatal and delivery care. Among the cohort of women, only two reported that they preferred traditional birth attendants while five reported that culture forbids the use of facility delivery. Also, the fact that being a Muslim in the LGAs (with multiple religious affiliations) increased the odds of PHC delivery suggests that religion is not an important deterrent, with all religious faiths in the two LGAs showing substantial use of health facilities. We believe that community health education can counter the effect of culture and tradition and increase the use of PHCs by rural women for skilled pregnancy care.

### Strengths and limitations

The major strength of the study is its community design approach using a representative sample of women in 20 predominantly rural communities in a geographical zone of Nigeria. This ensures that the results can be generalized to the entire Nigerian healthcare system, especially for rural communities in the country. The study was also conducted by a trained research team that was embedded in the project communities over 17 days. This enabled confidence building with community members, ensuring accuracy and reliability of data collection. Our use of CAPI ensured further accuracy and speed in data collection, enabling the generation of the data and related statistics immediately after the field work. This ensures the currency of information needed to design interventions for improving the access of rural women to skilled pregnancy care.

The major limitation of the study is our inability to collect information from recently delivered women on use of antenatal care. This was possibly due to difficulty in recalling events related to antenatal care which predate the time of delivery. While women could recall delivery events, they were less able to recall antenatal care events possibly due to better inclination to recall the outcomes rather than the processes of pregnancy care.

Despite this limitation, we believe that the results of this study are useful for designing interventions to improve the delivery of PHCs for skilled pregnancy care in Nigeria. Incidentally, the Federal Ministry of Health and all States of the country have identified PHC as the primary instrument and target for improved universal coverage that would ensure skilled pregnancy care for the reduction of maternal mortality in the country [38]. We believe that the results of this study are useful for scaling evidence-based interventions for alleviating the demand and supply factors that hinder the access of rural women to skilled pregnancy care in the country.

## Conclusion

We conclude that considerations for distance and costs, and perceptions relating to poor quality care are the factors that mostly hinder women’s access to skilled pregnancy care in PHCs in rural Nigeria. Efforts devoted to addressing these factors using innovative approaches will likely increase pregnancy care utilization in PHCs and reduce maternal mortality in rural Nigeria.

## Abbreviations

LGA: Local government area
NPC: National population commission
NPHCDA: National primary health care development agency
PHC: Primary health care
UNFPA: United Nations fund for population activities
WHO: World health organization
